# A light-efficient and versatile multiplexing method for snapshot spectral imaging

**DOI:** 10.1038/s41598-024-66386-2

**Published:** 2024-07-12

**Authors:** David Andersson, Yupan Bao, Vassily Kornienko, Dean Popović, Elias Kristensson

**Affiliations:** 1 Division of Combustion Physics, Department of Physics, Lund University, Professorsgatan 1, 22363 Lund, Sweden; 2 Institute of Physics, Bijenička cesta 46, 10000 Zagreb, Croatia; 3 Present Address: N2 Applied, Dronning Eufemias Gate 20, 0191 Oslo, Norway

**Keywords:** Multiplexing, Imaging, Multispectral, Imaging and sensing, Imaging techniques

## Abstract

The study of rapid and stochastic events that involve multiple species, such as chemical reactions and plasma dynamics, requires means to capture multispectral information in two dimensions at both high temporal- and spatial resolution. Commercially available cameras that provide high temporal resolution are based on either signal intensification or rapid data acquisition. Intensified cameras provide extremely short acquisition times using intensification by means of micro channel plates, but the conversion between electrons and photons makes these cameras inherently monochrome. In contrast, high-speed cameras can achieve color-sensitivity through integrated Bayer filters but suffer from a reduced light collection efficiency and a fixed spectral composition. In this article we present a non-integrated optical arrangement for instantaneous multispectral imaging based on FRAME image multiplexing. By spectrally separating the signal using lossless dichroic mirrors, a 16-fold increase in light-collection efficiency is gained (compared to past solutions), resulting in an equivalent increase in temporal resolution. This improvement provides new avenues for multispectral imaging of rapid events. We demonstrate the system’s versatility and suitability for studies of such processes by applying it for (i) temperature mapping using a high-resolution CCD camera, (ii) high-speed videography up to 10 kHz at four spectral channels and (iii) dual-species visualization in a plasma discharge using an intensified sCMOS camera.

## Introduction

Multispectral imaging is an invaluable tool with many applications in research areas such as medicine^1^, biology^2^, material sciences^3^, astronomy^4^, and agriculture and environmental studies^5–7^, due to its ability to simultaneously create a spatial and spectral profile of an object. In these applications the focus is often on computer vision and more specifically semantic segmentation, a task which in recent years have come to be dominated by machine learning approaches and big data^8^. To this end, focus is often on gathering as much unique information as possible in order to facilitate machine learning, and better resolved spectral information is one way of increasing the amount of information collected. A plethora of different technologies exist in order to be able to capture such information^9–13^.

A rudimentary method of capturing multispectral images is to use sequential acquisitions for each color channel^9^. By taking a series of images using a monochrome sensor and changing color filter in between acquisitions (through the use of e.g. a mechanical filter wheel), a series of images representing different color channels can be captured of an object. These can then be compiled in post-processing into a composite color image, with timescales on the order of $10^{-2}$ s between images (ASI Imaging FW-1000, Thorlabs FW103H). The benefit with this type of optomechanical solution is that the full spatial resolution of the sensor is preserved and spectral bands can easily be purpose-fitted, but it is unsuitable for use within research on fast, transient events due to its slow acquisition rate. The acquisition rate can be increased by using multiple sensors in parallel^10^, one dedicated for each specific color channel—an expensive solution, in particular when intensified cameras or high-speed are required.

On the other hand, multispectral imaging through the use of color filter arrays (*CFAs*) creates a periodic mosaic of a preset number of color filters (corresponding to the number of color channels), which is then physically superimposed onto the sensor. This results in several sparser color images being collected synchronously, which are then combined into a composite color image using a demosaicing algorithm. CFAs generally refer to filter arrays of three to four color channels in the visible range, but many filter patterns for more highly resolved spectral information, so called multispectral filter arrays (*MSFAs*) have been developed^11,14–16^. The main drawbacks of methods based on CFA technology (from a multispectral imaging perspective) is that (1) it relies on discarding light, with a reduction of the Signal-to-Noise ratio (*SNR*) that grows with the number of color channels and (2) any scientific filter (e.g. band-, low- or highpass filter) being applied on the camera will affect all channels. Recent work in the field of metasurfaces^12^, where spectral filter arrays are replaced by sub-wavelength structure arrays that interact with the electromagnetic field, are showing promising improvements with higher transmission rates. However, all filter arrays are manufactured on a laborious and end-user purpose basis and thus lack the ability to change spectral bands, as that would require changing the design of the metasurface.

An alternative approach to acquire multispectral information in 2D is to use image multiplexing schemes^17–22^. The general principle for this concept is to imprint a fine and unique spatial variation—a “fingerprint”—onto the intensity profile of the different spectral images. All spectral images are then captured simultaneously by the sensor, and are analytically separated in the post-processing using the encoded “fingerprints”. Several spectral multiplexing techniques have been published, utilizing different types of spatial intensity variation patterns and decoding methods. In 2008, Wagadarikar et al. demonstrated a multispectral imaging approach referred to as coded aperture imaging^17^. To multiplex the spectral components, the signal emitted by the target is first superimposed multiplicatively with a random binary pattern and thereafter continuously smeared along one axis using a light-dispersing device, e.g. a prism^17^ or grating^18^. The coded light is then detected and decoded into a hyperspectral image dataset using a compressed sensing algorithm^23^. While coded aperture imaging provides excellent spectral resolution—dictated by the spatial resolution of the grid pattern and the detector—the spatial overlap of the coded light leads to a similar problem as for CFAs, namely that any spectral filtering applied to the signal prior to its detection will affect all spectral channels. This intrinsic coupling between all spectral channels makes, for example, balancing of differently intense color components challenging.

An alternative to random binary patterns is to use two-dimensional spatial frequency patterns to encode and multiplex spectral image information^19–21^. This approach relies on encoding the spectral components individually, either through spatial- or temporal separation, in contrast to coded aperture imaging where the encoding occurs prior to any spectral dispersion/separation. In 2020, Dorozynska et al. demonstrated an optical arrangement using spatial frequency encoding, referred to as FRAME (Frequency Recognition Algorithm for Multiple Exposures), capable of snapshot multispectral imaging^22^. In the setup, the light emitted by the target was (i) split into four spatially separated optical paths, (ii) optically filtered, (iii) guided through a transmission grating of a unique spatial frequency and (iv) spatially recombined before detection. The spectral components were then digitally separated in post, by means of a spatial frequency lock-in algorithm. While spatial frequency encoding, in general, provides fewer spectral images than coded aperture imaging does, the setup demonstrated by Dorozynska et al. allows for channel-specific filtering, where each spectral channel can be optically modified independently. This feature becomes essential in applications where only particular wavelength regions are of importance, or where the spectral composition exhibits significant variations in intensity and requires balancing. However, while this spatial light-splitting strategy, based on optical beam-splitters, grants a high degree of control, it comes at the cost of light efficiency compared to the dispersion-based division of light associated with coded aperture imaging.

In this paper we present an improved optical arrangement for FRAME-based multispectral imaging using lossless light-splitting optics, yielding a 16-fold increase in light efficiency. The system is, in principle, not restricted to any type of imaging sensor technology, allowing for applications within many different scientific areas and imaging conditions. We demonstrate this versatility as well as the increase in temporal resolution directly gained by the improved light efficiency by combining the system with (1) a standard high-resolution CCD camera to quantitatively measure temperature in 2D, (2) a high-speed camera in order to gain multi-spectral 2D information at 10 kHz and (3) a time-gated intensified sCMOS camera to spectrally map different species in a plasma jet on a nanosecond time-scale.

## FRAME

**Figure 1:**
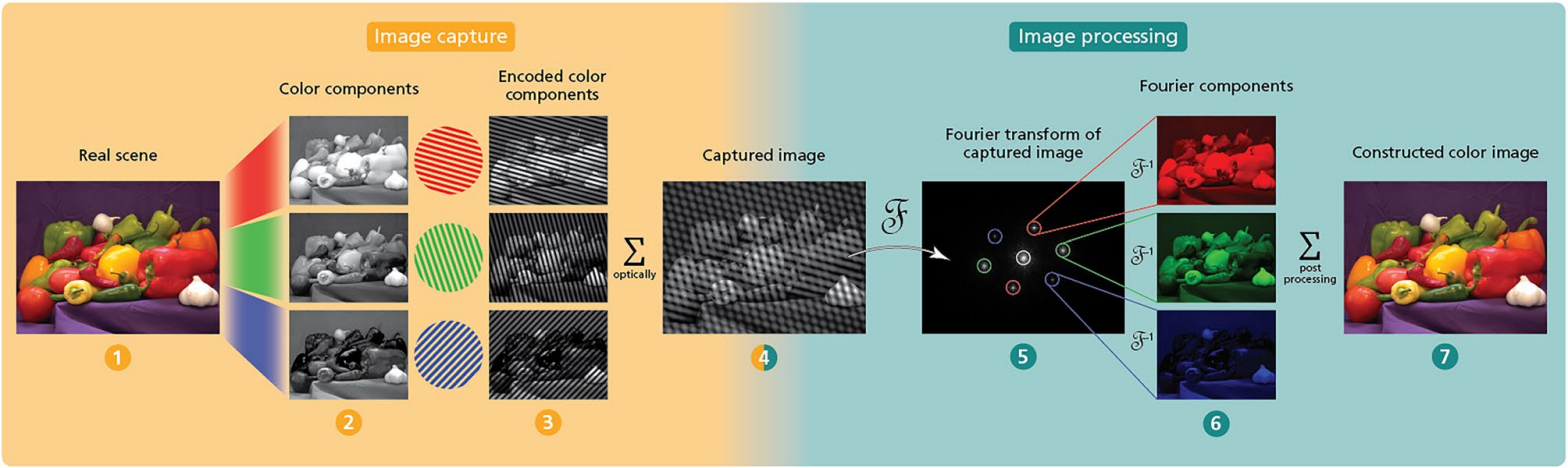
Schematic overview of the FRAME spectral multiplexing process. A colorful scene (1) is to be imaged in color using a monochrome camera. Before the light is directed into the camera, it is split into a number of optical channels, in this case three—red, green and blue (2). In each of these optical channels, an intensity modulation is introduced in the form of a unique spatial frequency, resulting in the modulated images seen in (3). The optical channels are then recombined and the resulting camera image (4) is produced. Once the image containing multiplexed spectral channels has been captured, each individual image can be extracted in post-processing. When applying the Fourier transform and viewing the raw image in the frequency domain (5), six frequency peaks can be observed, corresponding to the carrier frequencies of the red, green and blue channel respectively. By locking into each one sequentially, low-pass filtering around it, and performing an inverse Fourier transform, each spectral channel can be isolated (6). The three spectral channels can then be used to create a color composite, recreating the scene in color (7).

FRAME is an image multiplexing method that captures several images in one single camera exposure. Figure 1 shows a breakdown of the steps taken in order to encode and extract each individual multiplexed image. This is done by tagging each image with a unique intensity modulation in the form of a two-dimensional spatial carrier frequency before capturing them all simultaneously. After capture, the individual images can then be separated by sequentially locking in to their carrier frequencies, as the information of each image will be isolated in the frequency domain. Since FRAME is a purely optical technique, it does not introduce any electronic limitations and can be combined with all types of focal plane arrays, (*FPAs*). By changing sensor type, the system can be used in many different types of imaging scenarios to enable the sensor to capture parameters otherwise unavailable to it.

FRAME is a multidimensional optical imaging technique, meaning the set of images extracted from the single camera image can be used to examine parameters such as temporal variation^24^, polarization states^25^, and spectral composition. Depending on the parameter of interest, the intensity modulation is imparted on the image in one of two ways—either by (1) modulating the profile of the light illuminating the target, referred to as *Active FRAME*, or by (2) modulating the light emitted or reflected from the target, here-after referred to as *Passive FRAME*. Although subtle, there is a clear difference between the two. Active illumination grants very accurate temporal control, limited by the illumination source rather than the sensor. On the other hand, Passive FRAME collects spatial information about emission spectrum and polarization state in a snapshot. The two methods are also compatible, enabling instantaneous spatial mapping of the excitation- and emission characteristics of a sample that contains several different fluorescent markers, as demonstrated by Dorozynska et al.^21^.

Multiplexing image information via FRAME is enabled by trading the spatial- and dynamic range bandwidth of the sensor. Spatial lock-in detection yields a loss in spatial resolution as the images share the spatial bandwidth offered by the sensor. The magnitude of this loss is thus dictated by the number of multiplexed images^26^. Similar loss in spatial resolution is, however, inherent for most, if not all, snapshot multispectral imaging devices, such as methods based on Bayer filters or compressed sensing. If the images have structures that spatially overlap in the imaged scene, the dynamic range of the extracted images is reduced by a factor of *N*, where *N* is the number of multiplexed images.

The work presented in this article expands on the multispectral possibilities of the Passive FRAME technique in high temporal precision scenarios.

## Experimental setup

### The optical multiplexing setup

**Figure 2:**
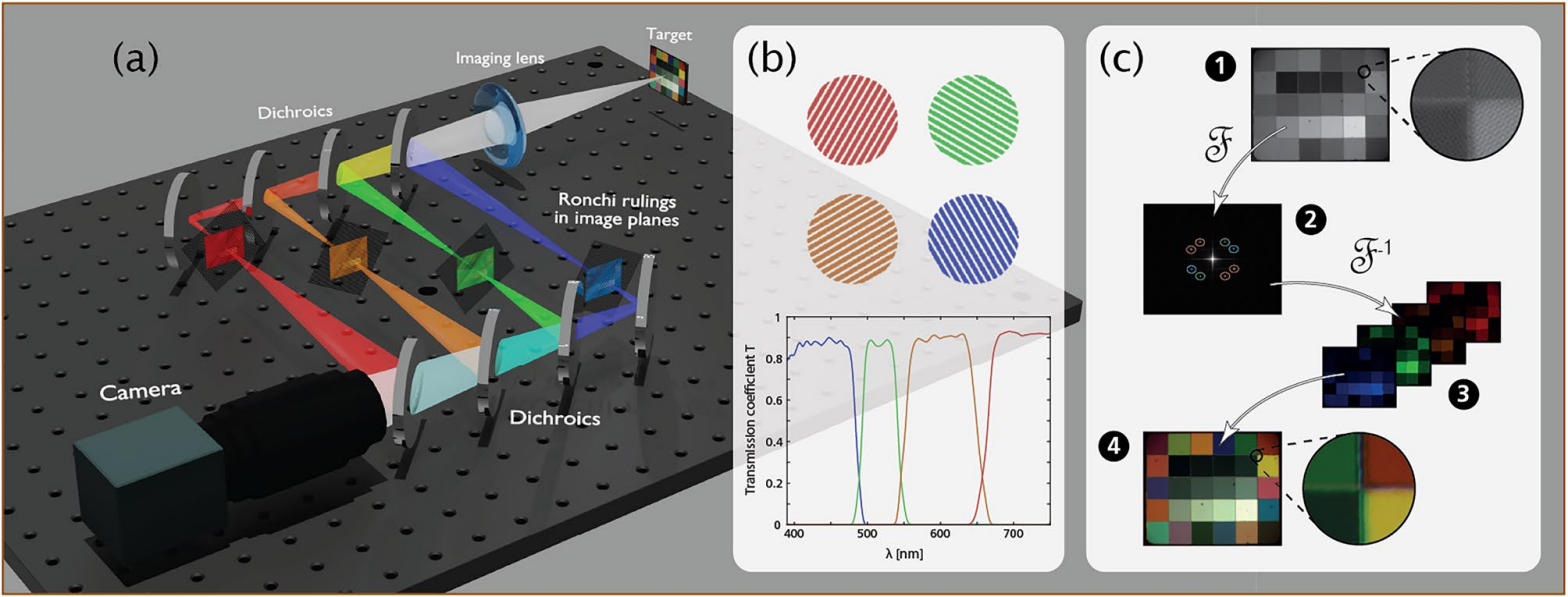
Optical setup and encoding/decoding procedure. The optical setup used for spectral multiplexing can be seen in (**a**), and utilizes dichroics in combination with Ronchi rulings in order to divide the spectral signals into different channels and impart spatial carrier frequencies onto the images. The inset (**b**) shows an example of how such carrier frequencies may look. In this case the magnitude of the frequency is the same, but the direction differs between channels. The transmission coefficient for each channel within the range 390–750 nm is also shown in (**b**), simulated using data provided by the optics manufacturer. Inset (**c**) shows the four steps of the decoding process—(1) the raw monochrome image captured contains the spatial frequencies from the Ronchi rulings, (2) due to these carrier frequencies, the discrete Fourier transform of the image contains frequency peaks shifted from the central frequency component, (3) isolating the information in these frequency peaks in turn isolates the information within a certain color channel, and (4) a multispectral image of the scene can be constructed through a linear combination of the color composites.

The optical setup used for spectral multiplexing can be seen in Fig. 2. The emission from the target is split spatiospectrally using dichroic mirrors (Thorlabs, DMLP/DMSP-series), creating four different spectral channels (390–490 nm, 490–550 nm, 550–650 nm, and 650–750 nm respectively). In each channel, a real image of the target is created using the imaging lens, and a Ronchi ruling with a unique rotational angle (and/or unique spatial frequency) is placed in the image plane to impart the intensity modulation. All four channels are then spatially overlapped and all Ronchi rulings with corresponding target images are simultaneously imaged onto the sensor by the camera lens, a Nikon macro camera lens (*f* = 105 mm) used in all experiments. The symmetry of the optical setup and the use of achromatic imaging lenses ensures equal optical path lengths in all channels. The intermediate image planes are thus formed equidistantly from the camera, providing equal foci and fields-of-view. The result is a grayscale image containing multiplexed spectral information by means of the intensity modulation patterns. Fourier analysis of this raw (unprocessed) image reveals frequency peaks corresponding to these carrier frequencies. The information carried by each individual frequency can then be isolated through a lock-in and de-modulating algorithm, resulting in four spectral images of the target, which can be used to construct a color composite.

The main difference compared to past optical solutions for multispectral imaging based on FRAME multiplexing^21,22^ is how the division (and encoding) of spectral information is achieved. Instead of splitting and recombining the light emitted by the sample spatially by means of beam-splitters combined with spectral filtering for wavelength sensitivity (each action yielding a loss of 75%), both these light-reducing actions are now carried out using the dichroic mirrors. The light-efficiency of the system is thus increased by a factor of $\sim$ 16, with a simulated light retention of 83% for the dichroic mirrors in the range 390–750 nm. Note that this calculation assumes a configuration based on four spectral channels, equipped with the same spectral filters used within the current experiment.

### Calibration of the multiplexing setup

For all quantitative and most qualitative measurements, it is necessary to perform a flat-field calibration of the imaging system to account for the differences in relative intensity arising due to the different optical pathways of the four channels. This calibration counters two main sources of error—(1) difference in average modulation depth (the contrast of the modulation) between different channels, causing relative intensities between these channels to be skewed, and (2) local differences in modulation depth in a single channel, causing errors in local intensities within a single multiplexed image. The first type of error can be introduced by, for example, a slight inaccurate positioning of the Ronchi rulings along the optical axis, leading to a reduced contrast of the imparted modulation in the plane imaged by the camera. Differences in local modulation depth within a single channel, causing the second type of intensity error, can arise if the Ronchi rulings are not aligned orthogonally with respect to the optical axis. These errors can be accounted for by performing a flat-field calibration using a multiplexed capture and a set of sequential captures as reference, taken through each individual optical channel, enabling quantitative measurements using spectral multiplexing.

### Two-dimensional temperature measurement

A high resolution monochrome CCD camera (ImperX B3420M, 9 megapixels) was mounted with the camera lens set to $f_\#$ = 8 and used with the optical multiplexing setup to estimate flame temperature by comparing relative intensities of emitted blackbody radiation in different spectral bands. To increase the accuracy of this type of thermography, band-pass filters were inserted into the three reddest channels (Edmund Optics 510 nm CWL/20 nm Bandwidth, Thorlabs FB600-10, Edmund Optics 655 nm CWL/40 nm Bandwidth). The system was calibrated and validated using a Schott KL 1500 LCD blackbody emulator source, capable of emulating blackbody radiation ranging from 2650 to 3300 K, and thereafter applied to a soot-rich propane diffusion flame produced by a Bunsen burner.

### High-speed imaging

To test the compatibility with high-speed imaging, the optical multiplexing setup was combined with a monochrome CMOS high-speed camera (Photron SA-5). During the experiment, the camera was run in 150K-capture mode at a shutter speed of 10 kHz with a spatial resolution of 768$\times$640 pixels. A LED array fixture was used to illuminate moving varicolored targets (fuse beads, 5 mm diameter hollow cylinders), with a field-of-view of approximately 20$\times$20 mm$^2$ in the object plane.

### Multi-species imaging

For multi-species imaging, an Andor iStar sCMOS-18U-63 camera was mounted to the optical multiplexing setup to image the spatial distribution of the plasma emission of two major species, ionized nitrogen (N$_2^+$) and excited helium (He I), of an atmospheric helium plasma jet. The same plasma jet has been studied previously and described in detail by Popović et al.^27^ with an applied voltage of 4 kV and pulse length of 10 $\mu$s. In this work, instead of a microsecond discharge, a nanosecond pulse generator (FID GmbH FPG 200-1NM1) which generates high-voltage pulses with a FWHM of 4 ns was applied. For results demonstrated in this paper, the repetition frequency of the pulse generator was set to be 1 Hz with four different voltage amplitudes, i.e., 30 kV, 40 kV, 50 kV and 60 kV. The plasma jet was placed 8.5 cm above a grounded stainless-steel plate and tested with three different Helium flow rates, i.e. 1.5, 3 and 4.5 SLPM (standard liter per minute). The f-number of the camera lens was set to $f_\#$ = 8, while the gate of the intensifier was set to 100 ns with 80% of the intensifier’s maximum efficiency.

## Results

### Validation of the optical multiplexing setup

**Figure 3:**
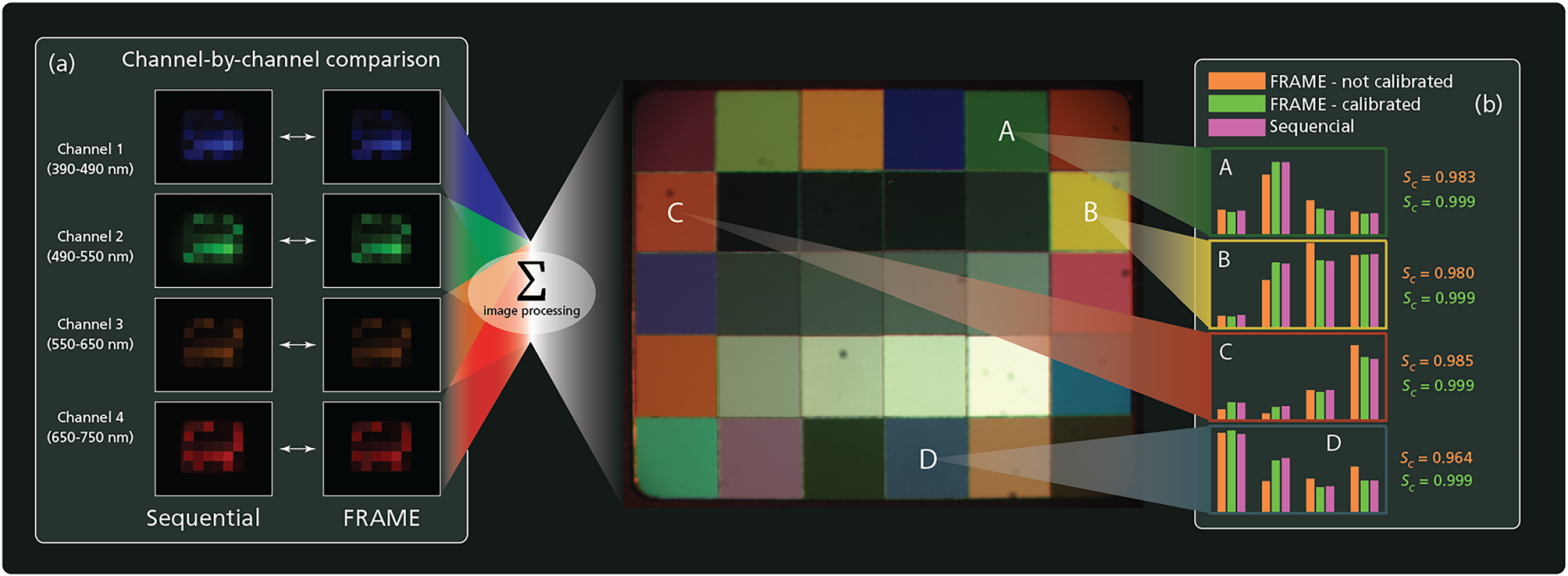
Comparison between sequential multispectral image acquisition and spectral multiplexing using FRAME. A color rendition chart containing various colored subregions has been reconstructed in color from multiplexed spectral channels captured in monochrome. The scene was captured through the presented optical system using both sequential imaging, where each color channel is captured independently and without Ronchi rulings, and through spectral multiplexing, where all channels are captured in snapshot in the same camera exposure and separated in post. (**a**) The four individual spectral channels captured using the two methods, with the left column containing the sequential set and the right column containing the multiplexed set after calibration. (**b**) The spectral base vectors from the indexed regions in the target for sequential (magenta) and multiplexing before (orange) and after (green) calibration, with associated cosine similarities between sequential and uncalibrated/calibrated results.

A comparison measurement was done to evaluate the performance of the spectral multiplexing compared to conventional sequential multispectral imaging through the same system. A color rendition chart (Macbeth, 41$\times$35 mm), depicted in Fig. 3 was used as a target for this measurement, illuminated by an LED array fixture and imaged by the camera (ImperX B3420M, 9 megapixel CCD) through the multiplexing optical arrangement. In Fig. 3, sequential acquisitions through each channel (with no Ronchi ruling inserted) are compared to images captured using FRAME multiplexing where all channels are captured in snapshot in the same camera exposure. The color channels extracted through lock-in in the multiplexed capture were then combined in post-processing to create the color composite. The quality of the multiplexing color rendering was evaluated by integrating the intensity over a colored area of the target, creating spectral base vectors for both capturing methods. To quantify the similarity of the spectral base vectors between sequential acquisition (${\textbf {s}}$) and multiplexing (${\textbf {f}}$), the cosine similarity,

1 $$\begin{aligned} S_C({\textbf {s}},{\textbf {f}}) = \frac{{\textbf {s}}\cdot {\textbf {f}}}{||{\textbf {s}}||~||{\textbf {f}}||}, \end{aligned}$$

was calculated for each colored subregion of the color test target. To demonstrate the impact of the flat-field calibration described above, both non-calibrated and calibrated results are included for the multiplexed capture. The visual comparison between the individual channels displayed in inset (a) reveals no significant difference, which is also supported by the cosine similarities for the calibrated multiplexing displayed in inset (b). The non-calibrated results displayed in (b) also highlights the necessity of the flatfield calibration in this measurement, as the color rendition would otherwise not be accurate.

### Quantitative temperature mapping using multispectral imaging

Thermography is a technique that uses multispectral imaging in order to spatially map temperature, and is an important application used in combustion diagnostics and numerous industrial applications. Since a blackbody radiates energy according to Planck’s law, the shape of the resulting spectrum is uniquely determined by its temperature. This manifests itself in the intensity distribution over the spectral channels of the imaging system, enabling two-dimensional temperature measurements by fitting the resulting intensity distribution of each pixel to Planck’s radiation law.

**Figure 4:**
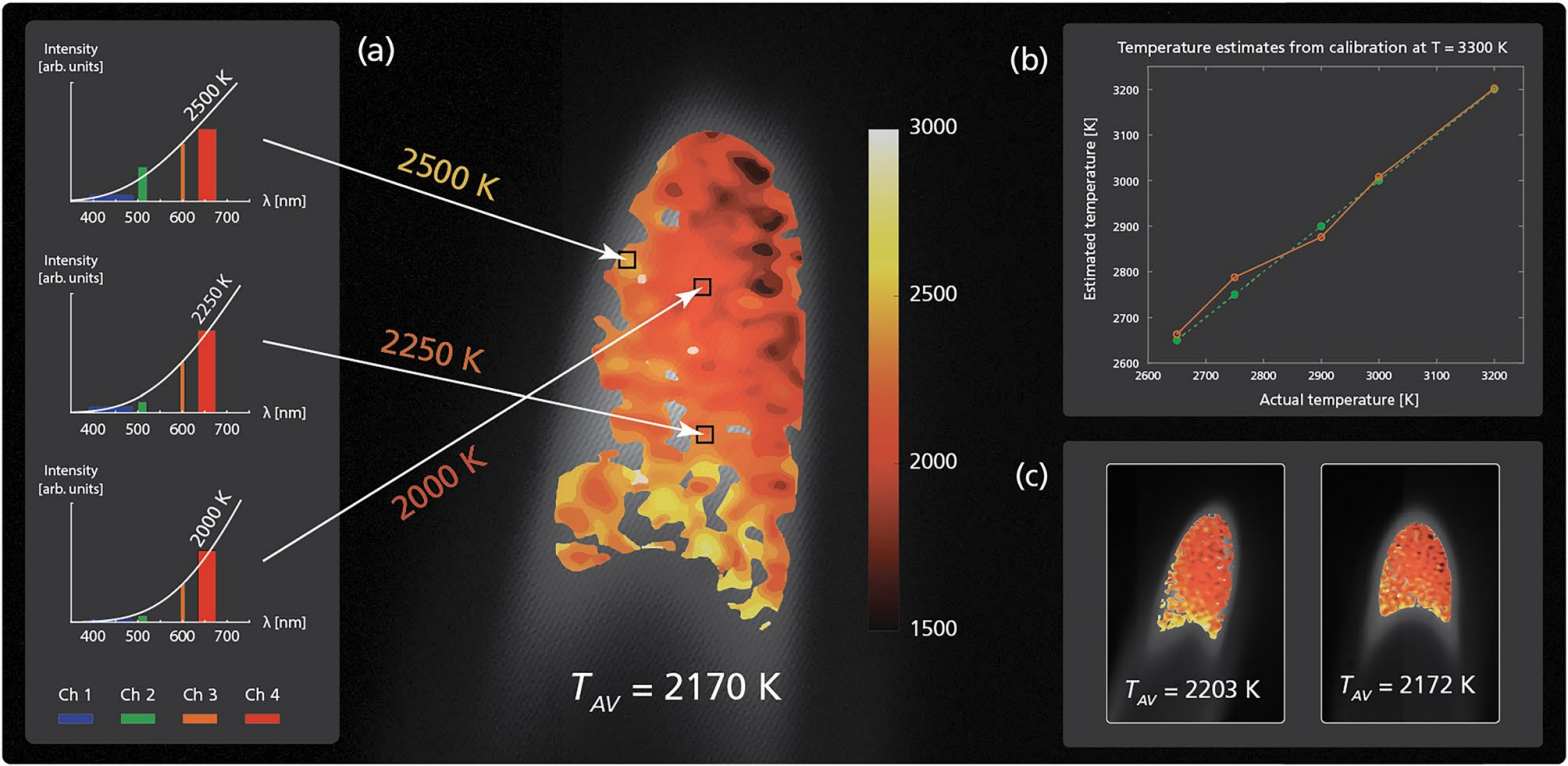
Thermography of a soot-rich propane flame. Four color composite images are captured in snapshot using spectral multiplexing on a CCD camera. (**a**) The spectral compositions of three different pixels and the Planck spectrum of their best temperature matching. This process is repeated pixel by pixel in order to produce the temperature mapping seen in the center figure, overlayed on the grayscale raw data. (**b**) A series of flatfield measurements performed in order to test the accuracy of the calibrated setup within a temperature range of 2650–3200 K. In (**c**), two further captures and their corresponding temperature mappings are showed, with all three having an average temperature of $\sim$ 2200 K.

Figure 4 shows thermography measurements of a propane flame performed in snapshot using spectral multiplexed imaging. The system is first calibrated against a flat-field blackbody spectrum of a known temperature, which is then compared to a simulated intensity distribution created using a perfect blackbody spectrum of the same temperature (allowing us to account for the effects that both optical components and modulation depth differences have on the relative intensities). When measuring on the flame, a snapshot multiplexed image containing four spectral channels is captured. Each pixel is then a four point resolved spectrum, and is compared to a library of simulated spectra of different temperatures to find the best match through cosine similarity. In order to exclude points not dominated by thermal radiation, which would otherwise give erroneous temperature readings, a minimum fit requirement has been set, leading to the blank areas in the thermal map. Note that the temperature range offered by the blackbody simulator used in the experiment and the extracted flame temperatures do not overlap, which may influence the accuracy of the extracted temperatures.

### 10 kHz multispectral sequence using high-speed camera

**Figure 5:**
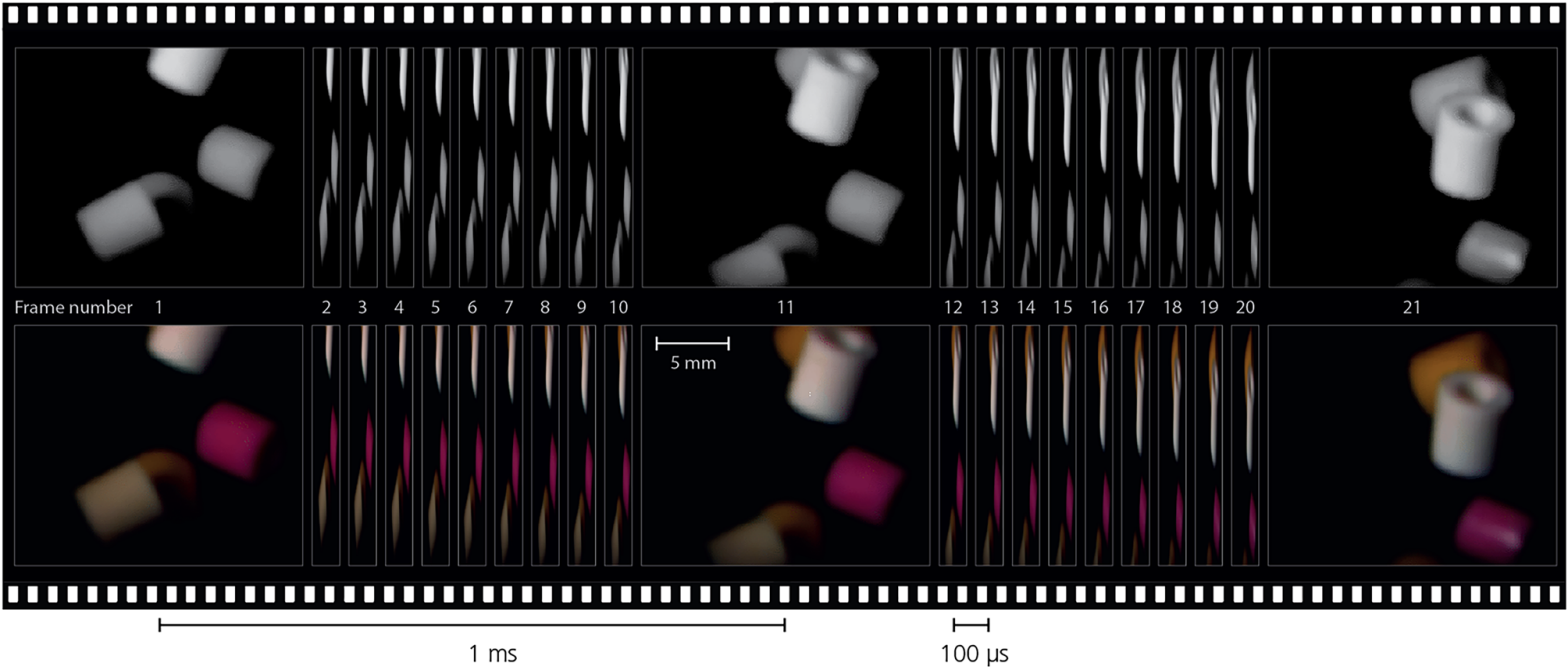
Image sequence captured in color at 10 kHz. In the experiment, a monochrome high-speed video camera was incorporated into the presented spectral multiplexing optical arrangement. The presented sequence contains 21 individual images covering a total duration of 2.1 ms, where each color image is the result of four color composites captured in snapshot through multiplexing. Since the color composites are all encoded into a single image, the technique does not impact the total sequence length possible in a measurement, which is dictated by the video camera. Frames 1, 11, and 21 are shown in full, while intermediate frames are compressed in the horizontal direction in the figure in order to compress the total size of the figure. Note that these intermediate images are still captured in the same resolution and aspect ratio, only displayed here in a compressed format. Top reel: monochrome images. Bottom reel: extracted color composites.

Rapid data acquisition cameras is a tool widely used within research to temporally and spatially resolve events occurring over timescales down to microseconds^28^. Such camera systems are most often constructed with monochrome capture in mind, in order to maximize the spatial resolution at a given capture rate, foregoing the capability to distinguish between different spectral components. This results in a technological gap for research requiring both spatial-, temporal-, and spectral resolution, most commonly solved by using multiple monochrome cameras combined with spectral filters. We present an optical arrangement solution, bypassing the need for dedicated multispectral high-speed cameras, by combining a monochrome high-speed camera with an image multiplexing arrangement. Figure 5 shows such a multiplexed sequence captured in monochrome at 10 kHz, where the scene has been recreated in color using the four color composites extracted from each raw image. This results in a maintained capture rate and field-of-view when compared to conventional monochrome capture. The difference in color helps in distinguishing targets that would otherwise be harder to separate (for example, the two lower left targets of the first frame). Some quantitative analysis can also be performed on this simple example—the speeds of the white and pink targets in center view can be calculated and compared. Adjusting for the difference in depth (position on the optical axis), the vertical speed of the two targets, assuming negligible speed in the focal direction, can be calculated to $\sim$ 3.7 m/s and $\sim$ 2.7 m/s for the white and pink target respectively. In this demonstration, the multispectral capturing helps distinguish between different targets, but the collection of spectral information can also be critical to a measurement, such as thermography or when identifying fluorescent markers or atomic and molecular species through emission spectra.

### Multi-species imaging of a pulsed plasma jet

Intensified cameras are, at present, the only 2D camera technology capable of sub-nanosecond time-gated imaging and are therefore essential in many different research fields. Unfortunately, these cameras are known to suffer from a degraded spatial resolution due to the inherent conversion between photons and electrons in the multi-channel plate^29^. To investigate whether such camera technology, despite this known degradation in image resolution, is compatible with Passive FRAME, simultaneous imaging of the plasma emission from two major species of a pulsed plasma jet has been performed with an intensified CMOS camera.

**Figure 6:**
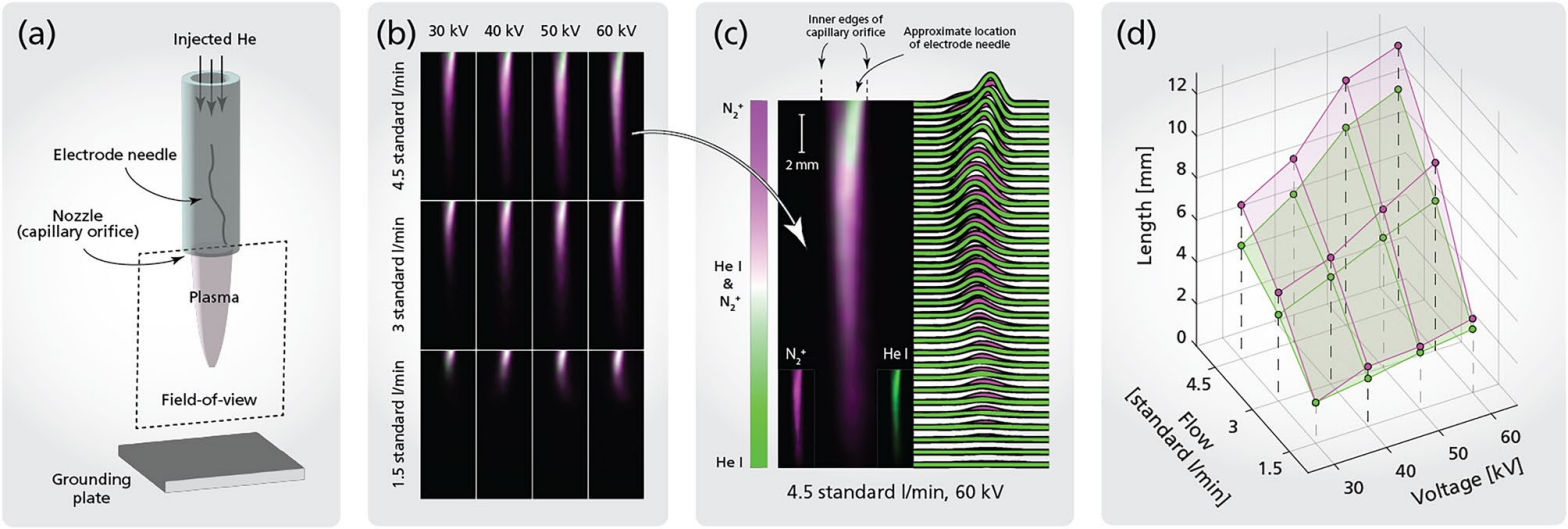
Snapshot imaging of He I and N$_2^+$ in a pulsed jet plasma. A schematic of the setup used to generate the pulsed jet plasma can be seen in (**a**). The two species are imaged simultaneously using spectral multiplexing for twelve different combinations of voltages (30, 40, 50, 60 kV) and flows (1.5, 3, 4.5 standard liter/min), and the resulting false-color images, with the two color channels depicting the two species, are shown in (**b**). (**c**) highlights the false-color image of the case 4.5 SLPM/60 kV, and also shows the plotted spatial profile of the pulsed jet plasma (intensity integrated over line-of-sight). (**d**) shows the length of the pulsed jet plasma (defined as when the intensity has decayed by 1/*e* from its maximum value along the ridge of the integrated intensity profile) as a surface spanned by the two varying parameters.

A sketch of the plasma jet and the field-of-view of the imaging system is shown in Fig. 6a. The same plasma jet has previously been studied by Popović et al.^27^, in which the optical emission spectrum is captured for an atmospheric pressure helium plasma jet with a longer pulse width (10 $\mu$s) and lower applied voltage (4 kV) than the nanosecond pulsed discharge (*NPD*) used in this work. Additionally, Jiang et al.^30^ has reported the plasma emission spectra of a similar helium plasma jet excited by two NPDs: one with an applied voltage of 8 kV and pulse widths of 5 ns, and another with pulse widths of 140 ns. Both studies^27,30^ observed plasma emissions predominantly arising from excited nitrogen below 400 nm, ionized nitrogen from 350 to 500 nm with peak intensity around 390 nm, and excited helium above 500 nm, albeit with differing relative intensities. Consequently, it is plausible to infer that the plasma emission recorded within channel 1 of the optical multiplexing setup presented in this work, 390–490 nm, originates from the first negative band of ionized nitrogen ($N_2^+$), while the plasma emission captured in channel 3, from 550 to 650 nm, is attributed to excited neutral helium (He I) emissions, mostly the 2*p* $^3P^0$–3*d* $^3D$ transition, i.e., the He I D3 line at 588 nm.

An example of the multi-species image of $N_2^+$ and He I after frequency lock-in analysis is shown in Fig. 6c. As the helium is injected into the air, it forms a conical volume filled almost exclusively with helium. The surrounding nitrogen, primarily excited by free electrons, is ionized mostly via Penning ionization effect by helium metastables^31^, resulting in a hollow profile. This hollow shape is observed in Fig. 6c as the outer boundary of the jet is dominated by nitrogen emission , while the inner core contains mainly helium. As the measurement is line-of-sight, the hollow structure of $N_2^+$ will not be observed in the ridgeline plot. The spatial profiles obtained show a high concentration of helium near the injection point (top-center of the image), due to the high flow of helium compared to the surrounding ambient nitrogen. However, emission from the surrounding nitrogen gradually starts to dominate downstream as the flow dissipates and the helium disperses. The spatial profile of the helium can be observed to be slightly tilted to the right-hand side in the top of the figure, caused by the electrode needle being slightly bent in this direction (see Fig. 6a). Since helium is primarily excited by collisions with electrons, the spatial profile of 588 nm emission should be mostly correlated to the electron density profile and neutral helium, hence the slightly noticeable inflection in the helium emission profile near the capillary orifice. As the measurement is line-of-sight, each pixel is in fact an integration along the optical axis of the system, meaning that even though the two species seem to spatially overlap in the projection of the image, they could be spatially separated along the optical axis.

False-color images of the $N_2^+$ and He I distribution at different locations downstream of the plasma jet is given in Fig. 6b for different operational conditions involving varying flow rates of helium and/or applied voltages. To study the impact of the flow rate and applied voltage on the plasma jet, the length of the plasma plume, defined as the distance at which the intensity of the plasma emission has decreased by a factor 1/*e*, for the tested operational conditions is further depicted in Fig. 6d. Notably, a higher flow rate or applied voltage leads to an elongated plasma plume. Interestingly, while the length of the plasma plume exhibits linear growth with increasing flow rate, its sensitivity to a higher applied voltage appears diminished. Moreover, a higher flow rate also results in a larger discrepancy in the length of $N_2^+$ and He I. However, such a systematic trend is not observed for higher applied voltages within the range of the tested operational conditions.

## Discussion

In this article we introduce a light-efficient optical arrangement for spectral two-dimensional multiplexing, capable of producing snapshot multispectral images with no decrease in camera capture rate. The implementation of dichroic elements in the splitting of the light into the optical channels and the subsequent recombination comes with two primary benefits. Firstly, a 16-fold increase in light efficiency is gained, compared to previously presented solutions using cubic beamsplitters and spectral filters, as the light is split (and combined) spatially on the criteria of wavelength, thus simultaneously performing the two actions previously done by separate components. The increased light collection in turn directly corresponds to an equal reduction in exposure time and thus opens up for applications with lower photon fluxes. Secondly, the number of multiplexed channels can now be freely adjusted in increments of one to suit the application, whilst maintaining its high light-efficiency. In contrast, in previous configurations the number of channels, *N*, can only be scaled in steps of $2^n$ (*n* $\in$ $Z+$), with a light-efficiency scaling as, at best, $1/N^2$. We validate the method and demonstrate its versatility as well as its applicability for low light conditions by performing both qualitative and quantitative measurements using three different FPA sensors—quantitative thermography with a high spatial resolution CCD, qualitative multispectral video sequence with a high capture rate CMOS, and qualitative multi-species imaging in a pulsed jet electric discharge (plasma) with an intensified sCMOS.

The possibility of turning any type of FPA into a multispectral device is a cost-efficient approach that opens up for many applications previously requiring multiple cameras or specialized equipment. The measurements in this work aim to demonstrate some of these possibilities, focusing on applications relying on short exposure times—an area usually confined to monochrome detection due to the relatively low efficiency that previously accompanied multispectral imaging. We further demonstrate the robustness and accuracy of our multiplexing imaging concept for both qualitative and quantitative measurements as well as the ability to actively balance different spectral components. This ability is an important aspect in order to maintain high sensitivity and dynamic range across a wide spectral range, and is unobtainable with multispectral methods based on Bayer filters or compressed sensing.

The main criteria for the application of our method is the ability to properly relay the image information through the spectral separation stage, without introducing image artifacts or aberrations. Yet, under such conditions, the method can be applied in many different imaging applications and is not bound to any specific configuration. For example, multispectral imaging is commonly used in microscopy to differentiate between different fluorescent markers and we believe our multiplexing concept can provide new means to target moving/living targets multispectrally within this area. Another possible application is long stand-off imaging, such as agricultural monitoring and astronomy, given that the loss in spatial resolution accompanying image multiplexing is acceptable. The parallel acquisition of specific spectral bands offered by our configuration may also provide new avenues for rapid diagnostic applications that rely on high-throughput, such as imaging flow cytometry and point (0D) measurements. In the case of point measurements, the spatial resolution of FPAs would provide means to acquire several multispectral point measurements simultaneously by using different regions in the FPA as individual boxcar integrators.

## Data Availability

The datasets used and/or analysed during the current study are available from the corresponding author on reasonable request.
