# Author Correction: A light-efficient and versatile multiplexing method for snapshot spectral imaging

**DOI:** 10.1038/s41598-024-80573-1

**Published:** 2024-11-26

**Authors:** David Andersson, Yupan Bao, Vassily Kornienko, Dean Popović, Elias Kristensson

**Affiliations:** 1 Division of Combustion Physics, Department of Physics, Lund University, Professorsgatan 1, 22363 Lund, Sweden; 2 Institute of Physics, Bijenička cesta 46, 10000 Zagreb, Croatia; 3 Present Address: N2 Applied, Dronning Eufemias Gate 20, 0191 Oslo, Norway

Correction to: *Scientific Reports* 10.1038/s41598-024-66386-2, published online 12 July 2024

The Funding section in the original version of this Article was incomplete.

“Open access funding provided by Lund University.”

now reads:

“Open access funding provided by Lund University. European Research Council (ERC) (803634). Swedish Research Council (2019-05183). Lund Laser Center is also acknowledged.”

The original Article has been corrected.

